# What fuels the employees in startups?: Data on hybrid/colocated/virtual working environment towards efficiency

**DOI:** 10.1016/j.dib.2023.109364

**Published:** 2023-07-14

**Authors:** Jenifer Esther D, Rashmi Rai

**Affiliations:** Christ (deemed to be University), Bannerghatta Road, Bengaluru 560076, India

**Keywords:** Startups, Job satisfaction, Workplace environment, Efficiency, Employees

## Abstract

**Purpose:**

This article examines the concepts of workplace satisfaction and productivity using data. The data will be used to investigate the variables contributing to employee satisfaction to achieve optimum efficiency through various startup working environments.

**Design/ Methodology/ Approach:**

Descriptive causal investigation. A structured instrument scale questionnaire via the internet to 256 employees working for highly organized organizations in Bangalore, India, using Qualtrics. The researcher adopted a simple random sampling method.

**Findings:**

The respondents in the data believed that the pre-covid workplace was advantageous. The hybrid model's prevalence of autonomy and flexibility increases work productivity. When employees are given more responsibility, their job satisfaction and productivity increase.

**Research Limitations/ Implications:**

Collecting data in a startup was extremely difficult due to the difficulty of obtaining permission, and through the analysis, it was determined that businesses have a responsibility to provide supplemental benefits to remote employees, which may increase the level of job satisfaction and enjoyment experienced by these individuals.

**Specification Table**

**Table:** 

Subject area	Business, Management, and Accounting
Specific subject area	Organizational Behaviour and Human Resource Management
Type of data	Table
How the data were acquired	Questionnaire, survey online and offline
Data format	Raw and Analysed
Description of data collection	Descriptive causal research. A structured instrument scale questionnaire was sent through the internet by Qualtrics to 256 employees working for organizations in Bangalore, India, that are highly organized. We used simple random sampling.
Data source location	• Institution: Tech Startups in Bengaluru • City/Town/Region: Bengaluru • Country: India
Data accessibility	Data is included in the link provided https://data.mendeley.com/datasets/3r5w5nmdvy/1/files/dc4021e8–383d-4e51-abe6–686cf5d60746

## Value of the Data

• Managers can use the data to support the process of knowing employee mode of work and how job satisfaction is based on performance efficiency. A combination of Job satisfaction and efficiency is analyzed with Startup data (see Ref. [2] for interpretations)
• The data can be used as a benchmark for research on employee workplaces in Startups. (For reimagining the workplace, see Ref. [1])
• The data can be used to compare the explanatory power of working remotely in startups, including the descriptive approach (for an example, see Ref. [6])
• The data can be used as teaching material.

## 1 Objective

To maximize productivity through various working environments in startups, the factors that affect employee happiness will be researched together with the data. Using the data, it has been determined that allowing employees to choose their working hours and tasks and where and when to perform them improves job satisfaction. Therefore, workplace autonomy increases job happiness.

The Covid'19 pandemic altered how almost all workers perform globally [3]. Remote work offers advantages and disadvantages. It facilitates commuting in flexible work settings. The Management of tasks, stress, and a lack of assistance are all challenges employers and employees ignore. Human resource professionals are concerned that the change may harm their employees' physical, emotional, and performance efficiency. Employee performance and benefits will increase if the issues are resolved [5]. The expansion of digital platforms has resulted in many tech startups [3]. Startups fail for a variety of reasons, and one of those causes is a personal one [4]. Therefore, it's crucial to meet their expectations for your task. So the researcher gathered data to find which work mode leads to job satisfaction.

## 2 Data Description

We examine the data collected by Qualtrics and the questionnaire designed using Instrument scales. The sample population consists of workers from Bangalore's established startups. The majority of information was gathered using Qualtrics-designed online survey. Even using telephone and staff interviews, fewer questionnaires were sent. The response rate is 60–70%. Minnesota Satisfaction Questionnaire (MSQ) produced a 20-item ``short form'' [7]. In this study, the abbreviated version of the original instrument was utilized to build the framework, and questions were divided into three domains: job efficiency, job expectation, and job satisfaction, comparing working circumstances before the COVID-19 pandemic to those during the pandemic. The reliability of the questionnaire and the data are measured through Jamovi and SPSS software, and it was found that Cronbach's alpha is 0.854. The questionnaire is reliable, and the survey is carried over to see the Post COVID'19 efficiency in the workplace.

The reliability of the questionnaire and the variables were analyzed, and the goodness of fit index and the p-value is <0.001, which is highly reliable. RMSEA is 0.144, the lower is 0.0914, and the upper value is 0.193 and the same is shown in Table 1. The data measured is both construct validity and discriminant validity. There is a positive relationship between similar variables.

**Table 1:** Factor analyses - output from Jamovi software.

Factor Loadings					
Factor	Indicator	Estimate	SE	Z	p
Factor 1	Q1_2	0.295	0.0802	3.68	< 0.001
	Q1_4	0.108	0.0868	1.25	
	Q2_2	0.273	0.0909	3.00	0.003
	Q2_4	0.151	0.0897	1.68	0.093
	Q2_6	0.168	0.0757	2.22	0.027
	Q2_7	0.113	0.0827	1.37	
	Q2_9	0.208	0.0729	2.85	0.004
	Q3_1	0.247	0.0856	2.89	0.004
	Q3_2	0.385	0.0750	5.13	< 0.001
	Q3_3	0.369	0.0731	5.04	< 0.001
	Q3_5	0.319	0.0832	3.83	< 0.001
	Q4	0.213	0.0686	3.11	0.002


Fit Measures				RMSEA 90% CI
CFI	TLI	RMSEA	Lower	Upper
0.665	0.591	0.144	0.0914	0.193

## 3 Experimental Design, Materials and Methods

Variables are separated and divided among a set of questions to make it easier to use the data for educational purposes and replication studies Under questions Q1_1 to Q1_4, the pre-covid section is discussed. The current working condition is covered in questions Q2_1 to Q2_Q9, and the impact of working remotely is wrapped in questions Q3_1 to Q3_5. Job satisfaction is discussed in Q4 to Q9 of this survey. Questions Q11_1 to Q11_5 are about the post-covid working expectation, while questions Q12_1, Q12_2, Q12_3, Q12_4, Q13_1, as well as Q13_2 are demographic variables in type.

### 3.1 Pre covid – Working Conditions

According to the data, five to six work days are too much for 65% of the sample. 69% of the model want their working hours reduced and their meetings emailed. Operating hours are too long for 70% of employees.

### 3.2 New normal

58% of telecommuters are more productive at home. Working from home saves time for half the data set. 72% of the model like working at home, and 82% want flexible hours. 81% prefer working from home to avoid commuting. 84% of employees say they spend more quality time at home, yet only 44% feel resourceful working at home.

### 3.3 Impact of working remotely in a conventional work setting

According to the data analysis, 70% of meetings are adequately performed, and 73% of employees can complete their tasks on time. 63% Employees may easily share work-related information on time. The problem with team cohesion exists because everything is done online, and 46% of the time, the personal touch is lacking.

### 3.4 Company response to COVID

Based on the data, it can be said that 86% of the time, the organization helped the workers do their jobs. About 17% of those who worked there did not get their full pay. More than half of the people in the sample are sure that their company doesn't have a backup plan in case of an emergency. However, 72% of the people feel safe in their job because 83% of them think the company keeps them motivated and asks about their overall health, and 97% of the organization has taken suitable COVID measures to ensure the workplace is safe.

### 3.5 Employees efficiency

The data shows that employees think working at the Office is more productive than working at home. 71.6% of workers think they work better in an office setting. Over 67.3% of people like a hybrid environment because they can choose between a job and home, depending on their needs. Also, 66.6% of employees think working from home is a good choice when there is no other way to work.

### 3.6 Post covid work expectation

From the post-COVID expectation part of the data set, we can see that companies expect employees to report to the organization, and the number of desired companies drops to 62%. 83% of employees prefer to work from home, and 93% are happy to work in a hybrid setup. 76% of workers will be satisfied if they only work four days a week instead of five. 89% of workers want to be able to set their hours.

### 3.7 Comparison between pre-COVID'19 and new normal (Post COVID'19) work expectation

From the data collected, it can be seen that 65% of employees at Pre Covid wanted the number of working days to be cut to four, and the number of employees who wished to have fewer working days went up to 76% when more employees worked from home. Before COVID, 69% of employees thought that in-office work hours would be cut down, and 89% of employees in the new normal(after COVID'19) expected more free work hours and were mainly happy to decide when they work.

Per Herzberg's two-factor theory of work happiness, we analyzed several aspects linked to hygiene and motivators to assess job satisfaction by thoroughly meeting job requirements. The data analyses through Jamovi software show that the study is statistically significant with the P value <0.001.

In line with the data analysis, it is significant that the statistical value of efficient working in the hybrid(mixed) way is 35.1- 20–40. T-test is 8–9% better than working remotely or in the Office because there are free to decide. It eventually leads to job satisfaction, and it can be substantiated that working at the Office is better to perform efficiently when compared to working from home.

Depending on the employee's age from the data, the working environment may affect job satisfaction differently. A link exists between working style, post-COVID employment expectations, and comparatively, working in an office setting results in more efficient performance than other modes.

Letting employees decide their working hours and task to complete the work by determining where and when to work leads to better job satisfaction. It is noted that pre covid working condition was satisfactory to some extent. The expectation of the post covid working condition, which leads to job satisfaction, is on flexibility in choosing a place and time. The statistic value is 30 using one Sample T-test, and the p-value is <0.001, which is the maximum. Hence, the flexibility to choose at ease is one of the essential motivators which leads to job satisfaction in the new normal era.

## Ethics Statement

The authors confirm that the provided dataset and presented work strictly meet the ethics requirements for publication in Data in Brief as mentioned in https://www.elsevier.com/authors/journal-authors/policies-and-ethics.

## CRediT authorship contribution statement

**Jenifer Esther D:** Conceptualization, Methodology, Validation, Writing – original draft, Data curation, Formal analysis. **Rashmi Rai:** Writing – review & editing, Supervision, Data curation.

## Declaration of Competing Interest

The authors declare that they have no known competing financial interests or personal relationships that could have appeared to influence the work reported in this paper.

## Data Availability

“What Fuels the Employees in Startups?”: Data on Hybrid/Colocated/Virtual Working Environment Towards Efficiency (Original Data) (Original data) (Mendeley Data).
